# Identification of characteristic metabolic panels for different stages of prostate cancer by ^1^H NMR-based metabolomics analysis

**DOI:** 10.1186/s12967-022-03478-5

**Published:** 2022-06-17

**Authors:** Xi Zhang, Binbin Xia, Hong Zheng, Jie Ning, Yinjie Zhu, Xiaoguang Shao, Binrui Liu, Baijun Dong, Hongchang Gao

**Affiliations:** 1 School of Pharmaceutical Sciences, Wenzhou Medical University, Wenzhou, 325035 China; 2 Department of Urology, Renji Hospital, School of Medicine, Shanghai Jiao Tong University, Shanghai, 200127 China; 3 Key Laboratory of Alzheimer’s Disease of Zhejiang Province, Institute of Aging, Wenzhou Medical University, Wenzhou, Zhejiang China; 4 Oujiang Laboratory (Zhejiang Lab for Regenerative Medicine, Vision and Brain Health), Wenzhou, 325000 China

**Keywords:** Prostate cancer, Biomarker, Serum, Metabolomics, Diagnosis

## Abstract

**Background:**

Prostate cancer (PCa) is the second most prevalent cancer in males worldwide, yet detecting PCa and its metastases remains a major challenging task in clinical research setups. The present study aimed to characterize the metabolic changes underlying the PCa progression and investigate the efficacy of related metabolic panels for an accurate PCa assessment.

**Methods:**

In the present study, 75 PCa subjects, 62 PCa patients with bone metastasis (PCaB), and 50 benign prostatic hyperplasia (BPH) patients were enrolled, and we performed a cross-sectional metabolomics analysis of serum samples collected from these subjects using a ^1^H nuclear magnetic resonance (NMR)-based metabolomics approach.

**Results:**

Multivariate analysis revealed that BPH, PCa, and PCaB groups showed distinct metabolic divisions, while univariate statistics integrated with variable importance in the projection (VIP) scores identified a differential metabolite series, which included energy, amino acid, and ketone body metabolism. Herein, we identified a series of characteristic serum metabolic changes, including decreased trends of 3-HB and acetone as well as elevated trends of alanine in PCa patients compared with BPH subjects, while increased levels of 3-HB and acetone as well as decreased levels of alanine in PCaB patients compared with PCa. Additionally, our results also revealed the metabolic panels of discriminant metabolites coupled with the clinical parameters (age and body mass index) for discrimination between PCa and BPH, PCaB and BPH, PCaB and PCa achieved the AUC values of 0.828, 0.917, and 0.872, respectively.

**Conclusions:**

Overall, our study gave successful discrimination of BPH, PCa and PCaB, and we characterized the potential metabolic alterations involved in the PCa progression and its metastases, including 3-HB, acetone and alanine. The defined biomarker panels could be employed to aid in the diagnosis and classification of PCa in clinical practice.

**Supplementary Information:**

The online version contains supplementary material available at 10.1186/s12967-022-03478-5.

## Background

Prostate cancer (PCa) is the second most frequent cancer among men globally, which poses a major detriment to men’s health [1]. There were an estimated 1.4 million new cases of prostate cancer, and almost 375,304 cancer deaths occurred in 2020 [2]. Several significant efforts have been focused on the development of sensitive and accurate diagnostic tools for PCa since it is more likely to be cured if it is diagnosed early [3]. Currently, PCa is majorly screened by the prostate-specific antigen (PSA) blood test or a digital rectal examination (DRE) combined with a subsequent ultrasound-guided prostate biopsy (PBs) that confirms the cancerous growth presence [4]. Several limitations still exist in the traditional diagnostic methods like, higher procedural costs, longer examination time, low sensitivity and specificity leading to false positive findings, which sometimes may lead to overdiagnosis and consequent overtreatment [5, 6]. Therefore, there is an urgent need to discover novel biomarkers for improving the diagnosis, prognosis and treatment of PCa.

As dysregulated cellular metabolism is a vital hallmark of cancer [7], employing metabolomics analysis in cancer samples could provide critical insights for monitoring the cancer progression [8, 9]. Recently, multiple studies have demonstrated the potential of metabolomics analysis in PCa research. For example, Lima et al. [10] analyzed metabolomic profiling of volatile metabolites in urine samples from PCa patients and healthy controls using a metabolomics approach and found that urinary signatures of volatile organic compounds and volatile carbonyl compounds allowed for accurate discrimination between PCa and control groups. Based on the liquid chromatography-mass spectrometry (LC–MS) and chromatography-mass spectrometry (GC–MS), Huang et al. [11] suggested that serum N-oleoyl taurine and sterol metabolites levels were linked to a decreased PCa survival rate. Additionally, a serum metabolomics analysis integrated with GC–MS and magnetic resonance spectroscopy (MRS) separated benign prostatic hyperplasia (BPH) from PCa subjects, two common conditions that gave rise to increased PSA levels, and identified acylcarnitines, glycerophospholipids, and arginine that could be used as potential diagnostic markers for separating BPH from PCa [12]. Hence, this concept can be put forth that metabolomics analysis has a strong potential as a precise diagnostic tool for exploring metabolic biomarker’s expression patterns and elucidating the potential PCa metabolic mechanisms [13].

Nuclear magnetic resonance (NMR) spectroscopy is a promising noninvasive technique for metabolic profiling analysis due to innate advantages like simple sample preparation, non-destructive analysis, and high reproducibility [14]. In our previous study, ^1^H-NMR based metabolomics approach was successfully applied to characterize the significant metabolic differences in tissue and biofluid samples in five different PCa stages [15] as well as prostate tissue samples in hormone-sensitive and castration-resistant PCa cases [16]. In the previous study, we identified a series of metabolic disturbances, including energy, amino acid, choline, fatty acid, and uridine metabolism in five different PCa stages [15], and amino acid, choline metabolism, and Warburg effect from hormone-sensitive to castration-resistant PCa [16]. However, little information is available about tumor progression and metastasis. The present study characterized the metabolic profiling in serum samples from PCa, PCa patients with bone metastasis (PCaB), and BPH subjects via a ^1^H NMR-based metabolomics approach. The aim of this study was (1) to identify metabolic alterations among PCa, PCaB, and BPH, (2) to investigate key metabolic pathways involved in the PCa and PCaB progression, (3) to elucidate the potential biomarker panels for differentiating among BPH, PCa and PCaB subjects.

## Methods, materials and participants

### Study Participants

This is a retrospective cross-sectional analysis of 187 participants included in Renji Hospital of Shanghai Jiaotong University from April 2014 to October 2018, including 75 BPH, 62 PCa, and 50 PCaB patients. All participants underwent PSA screening, DRE, trans-rectal ultrasound (TRUS) and sometimes biopsy according to the PCa National Comprehensive Cancer Network (NCCN) guidelines [17]. All subjects were enrolled based on clinical criteria for different diagnoses of PCa and BPH by an urologist. The PSA values of all subjects were higher than 4 ng/mL. PCa patients were defined by the following criteria: (1) nodules at DRE; (2) hyperechoic or hypoechoic lesion at TRUS; (3) biopsy-proven prostate cancer (Gleason score for the biopsy > 6). Additionally, PCaB patients were defined with distant bone metastases by X-rays and bone scan analyses. BPH patients were diagnosed after a negative DRE, TRUS or biopsy evaluation to rule out PCa. The study protocol was approved by the Ethics Committee of Shanghai Jiao Tong University School of Medicine (IRB number: Renji [2013] 26) along with the informed written consents obtained from all the participants. The detailed pathological and clinical subject parameters are listed in Table 1.

**Table 1:** Participants’ pathological and clinical characteristics

Parameters		BPH^b^	PCa^c^	PCaB^d^	p value^a^		
					PCa vs BPH	PCaB vs BPH	PCaB vs PCa
N		75	62	50	–^e^	–	–
Age (year)		65.15 ± 7.46	68.53 ± 7.58	73.82 ± 8.17	0.009	< 0.001	< 0.001
BMI (kg/m^2^)		24.10 ± 2.56	24.62 ± 2.56	23.38 ± 2.90	0.249	0.129	0.014
PSA (ng/mL)		12.64 ± 5.27	18.51 ± 15.92	401.16 ± 124.46	0.058	0.024	0.027
Gleason score	3 + 3	–	17	3			
	3 + 4	–	11	8			
	3 + 5	–	0	2			
	4 + 3	–	22	10			
	4 + 4	–	8	18			
	4 + 5	–	2	6			
	5 + 3	–	1	0			
	5 + 4	–	1	2			
	5 + 5	–	0	1			

Data were presented as Mean ± SD

^a^The p values were calculated by using unpaired Student’s t-test with Bonferroni adjustment, the difference was considered to be statistically significant when p < 0.05

^b^BPH, patient with benign prostatic hyperplasia

^c^PCa, prostate cancer

^d^PCaB, prostate cancer with bone metastasis

^e^No data.

### Sample collection and preparation

Once accepted and enrolled into our study, body mass index (BMI) of subjects were recorded and blood samples were collected. All the participants were overnight-fasted and the fasting blood samples were collected on the next morning following a standard operating procedure. Then, the blood samples were thawed for 15 min under ambient temperature, and centrifuged for 10 min at 1024 g and 4 °C to obtain serum samples. Serum supernatants were collected and stored at –80 °C for further analysis. Before undertaking the metabolomic analysis, serum samples were thawed at 4 °C and vortexed for ten seconds, followed by the dilution of the serum sample (200 µL) with phosphate buffer (250 µL, 0.2 mM Na_2_HPO_4_/NaH_2_PO_4_, pH = 7.4) as well as deuterium oxide (D_2_O, 50 µL). Thereafter, the mixed serum sample was vortexed for ten seconds and centrifuged at 10,000 g for 15 min at 4 °C followed by the supernatant transfer (500 µL) into a 5-mm NMR tube for further metabolomics analysis.

### ^1^H NMR-based metabolomics analysis

^1^H NMR-based metabolomics analysis was performed on a Bruker AVANCE III 600 MHz NMR spectrometer (Bruker BioSpin, Rheinstetten, Germany) equipped with a five mm Triple Resonance Probe (TXI) probe to obtain the information about small molecule metabolites in serum samples. The ^1^H NMR spectra of serum samples were acquired by using a Carr-Purcell-Meiboom-Gill (CPMG) pulse sequence with a fixed receiver-gain value at 37 °C for negating the comprehensive effects of lipid or protein macromolecules. The main NMR acquisition parameters, as considered in our previous study [18], were determined as follows: data points = 256 K; relaxation delay = 4 s; spectral width = 12,335.5 Hz; acquisition time = 2.66 s per scan. All CPMG spectra were transferred to TopSpin 3.0 software (Bruker BioSpin, Rheinstetten, Germany) for automatic phase and baseline corrections while the chemical shifts of the serum spectra were directed toward the anomeric signal of α-glucose at 5.23 ppm. The icoshift algorithm based on spectral interval’s calibrations was applied to align NMR spectra in MATLAB (R2012a, The Mathworks Inc., Natick, MA, USA) [19]. Henceforth, the spectral region from 0.0 to 9.0 ppm, excluding the water peak from 4.70 to 5.00 ppm, was subdivided and integrated into binned datasets with sizes of 0.01 ppm and 0.0015 ppm for multivariable and quantitative analyses, respectively. Accordingly, the metabolites were assigned by using Chenomx NMR suite 7.7.2 software (Chenomx Inc., Alberta, Canada) and the Human Metabolome Database (HMDB) [20]. The concentrations of serum metabolites were calculated according to their peak area by reference to the total area of each NMR spectrum and expressed as relative units (r.u.).

### Multivariate analysis

This study conducted multivariate data analysis by using SIMCA 12.0 software (Umetrics AB, Umea, Sweden) that utilized partial least squares discriminant analysis (PLS-DA) to obtain a metabolic pattern discrimination overview among BPH, PCa and PCaB subjects. Furthermore, the orthogonal partial least squares discriminant analysis (OPLS-DA) model, another supervised method, was used to construct a group-based model for optimizing classification by removing unrelated variables of the class. The important metabolites for classification were identified by corresponding variable importance in the projection (VIP) scores, whereas metabolites with a VIP value > 1 were selected for further analysis. The OPLS-DA model’s quality was evaluated by cross-validated analysis of variance (CV-ANOVA), in which higher values of R^2^ and Q^2^ were considered as a goodness of fit and goodness of prediction parameters, respectively. The value of R^2^ and Q^2^ close to 1.0 indicates a good performance of OPLS-DA model. The principal component analysis (PCA) model was utilized to examine metabolic changes among different regions of age and BMI for detecting the influence of potential covariates on serum metabolome.

### Statistical analysis

Statistical analyses were performed using SPSS 22.0 software (IBM Corp, Armonk, NY). The unpaired Student’s t-test with Bonferroni adjustment was applied to measure the significance of each metabolite, age, BMI and PSA levels. The p values across all metabolites within each comparison were also adjusted to account for multiple testing by a false discovery rate (FDR) method. A FDR value of < 0.05 was considered statistically significant. Our study selected metabolites with VIP > 1 and FDR value < 0.05 and considered them as the significant differential metabolites that could discriminate within the groups for an accurate disease assessment. Metabolic pathways were manually drawn by CorelDRAW Graphics Suite (Corel Inc., Ottawa, Canada) based on KEGG pathways (www.genome.jp/kegg/) and Small Molecule Pathway Database (SMPDB, www.smpdb.ca/).

### Receiver-operating characteristic (ROC) analysis

Receiver operating characteristic (ROC) curves analysis was used to evaluate the diagnostic potential of variable-identified metabolites among BPH, PCa, and PCaB groups along with the generation of the area under the ROC curve (AUC) value by MedCalc software (MedCalc Software, Ostend, Belgium). The significance level was defined at a p value < 0.05, and the AUC value < 1 indicated excellent diagnostic accuracy.

## Results

### Participants’ clinical characteristics

A total of 187 subjects, including 75 BPH patients, 62 PCa patients, and 50 PCaB patients, were included, and their clinical characteristics are presented in Table 1. In the cohort, the mean age of patients was significantly greater in PCaB, followed by PCa and BPH subjects. Although BMI of PCaB patients was distinctly decreased compared to PCa, no significant alteration was observed in PCa and PCaB patients compared to BPH subjects. Furthermore, PCaB patients had distinct higher PSA levels than PCa and BPH groups; however, no difference was observed in PSA levels between BPH and PCa cases. Hence, the traditional detection based on PSA levels is inapplicable for separating BPH from PCa.

### Metabolic segregations among BPH, PCa, and PCaB patients

**Fig. 1:**
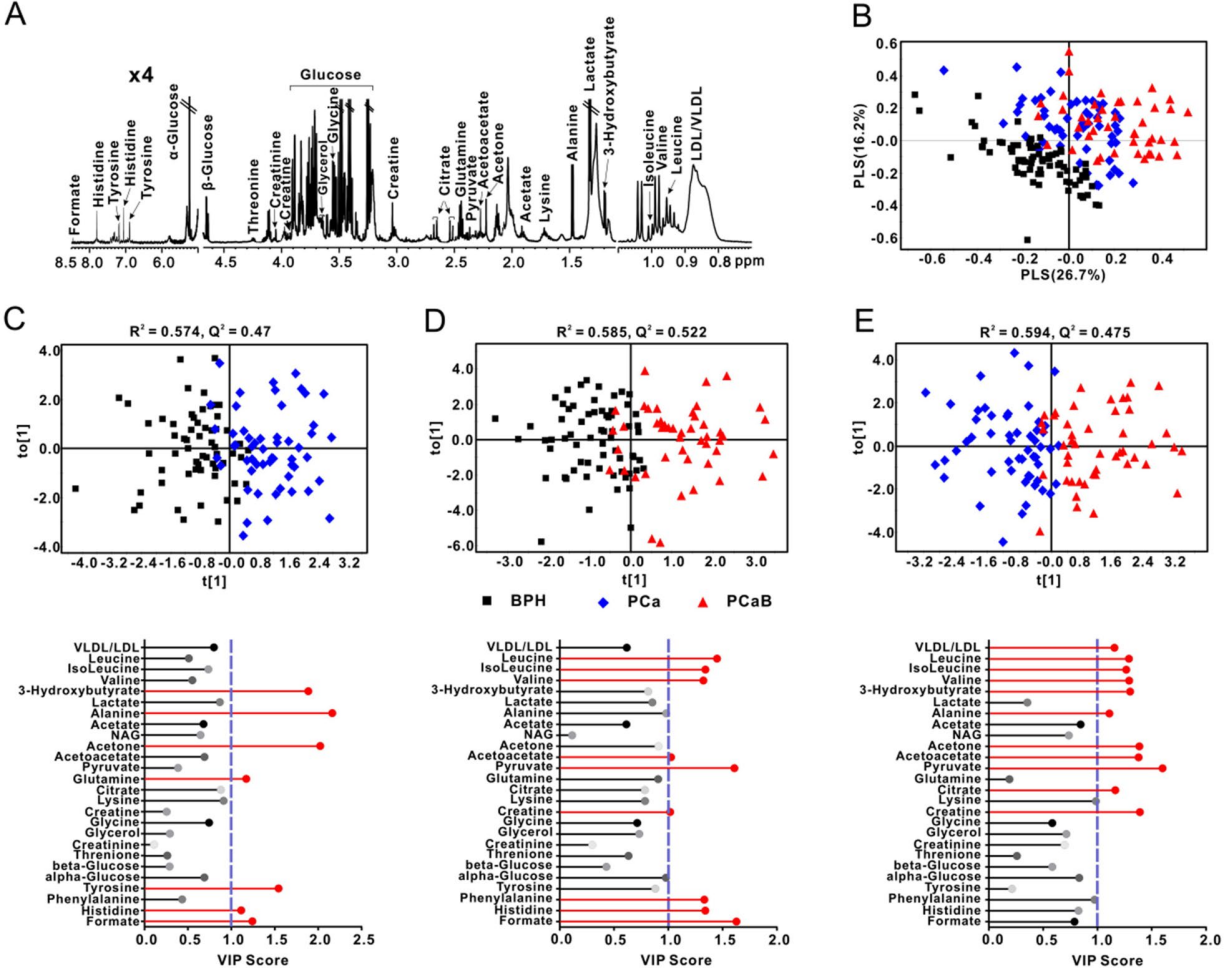
^1^H NMR-based metabolomics analysis. **A** Representative 600 MHz ^1^H NMR spectra obtained from BPH serum samples; **B** PLS-DA-based metabolic pattern discrimination among BPH, PCa and PCaB; OPLS-DA model-based classification between **C** BPH and PCa, **D** PCaB and BPH, **E** PCa and PCaB as well as corresponding VIP scores

This study comprehensively analyzed the metabolic profiles of serum samples obtained from 75 BPH, 62 PCa, and 50 PCaB patients by a ^1^H-NMR based metabolomics approach. Figure 1A illustrates a typical ^1^H-NMR spectrum of BPH serum sample containing 25 metabolites that were mainly involved in energy metabolism (citrate, creatine, creatinine, α-glucose, β-glucose, lactate, and pyruvate), amino acid metabolism (alanine, glutamine, glycine, histidine, isoleucine, leucine, lysine, phenylalanine, threonine, tyrosine, and valine), ketone body metabolism (3-hydroxybutyrate (3-HB), acetone and acetoacetate), lipid metabolism (LDL/VLDL and glycerol) and fatty acid metabolism (acetate and formate).

The PLS-DA, based on the serum metabolome characteristics, was carried out for determining the metabolic pattern changes among BPH, PCa, and PCaB groups. As illustrated in Fig. 1B, the PLS-DA score plot revealed a clear metabolic pattern demarcation between BPH, PCa, and PCaB subjects. Subsequently, a supervised OPLS-DA model was utilized for characterizing the discrimination between the two groups as well as the VIP score, which confirmed the differential metabolites. As can be seen from Fig. 1C–E, the discriminant capabilities of the PLS-DA models were observed in the following groups: between BPH and PCa (R^2^ = 0.574, Q^2^ = 0.47) (Fig. 1C), between BPH and PCaB (R^2^ = 0.585, Q^2^ = 0.522) (Fig. 1D) as well as between PCa and PCaB (R^2^ = 0.594, Q^2^ = 0.475) (Fig. 1E). The BPH and PCa segregation as displayed by the VIP score of OPLS-DA was mainly attributed to metabolites like 3-HB, alanine, acetone, glutamine, tyrosine, histidine, and formate (Fig. 1C). In contrast, significant metabolites like leucine, isoleucine, valine, acetoacetate, pyruvate, creatine, phenylalanine, histidine, and formate played a remarkable role in metabolic differentiation between BPH and PCaB patients (Fig. 1D). Additionally, LDL/VLDL, leucine, isoleucine, valine, 3-HB, alanine, acetone, acetoacetate, pyruvate, citrate, and creatine were also associated with PCa and PCaB dissociation in the serum metabolome (Fig. 1E).

Given that age and BMI were significantly different among BPH, PCa, and PCaB groups, we utilized a principal component analysis (PCA) model to examine the metabolic changes among different regions of age and BMI for detecting the influence of potential covariates on serum metabolome. As shown in Additional file 1: Fig. S1, there is no distinct separation of serum metabolome detected among different regions of age (see Additional file 1: Fig. S1A) and BMI (see Additional file 1: Fig. S1B), indicating that serum metabolome of BPH, PCa and PCaB pateints had a graded relationship with disease status that was not caused by age, BMI. Hence, we set aside the effect of different age and BMI on serum metabolome in the subsequent analysis.

### Quantification of differential metabolites among BPH, PCa, and PCaB

Statistical analysis of detected metabolites was obtained for exploring the characteristic metabolic changes among BPH, PCa, and PCaB groups in Table 2. When compared with BPH, PCa had significantly lower levels of 3-HB, acetone, glutamine and histidine as well as higher serum levels of alanine and tyrosine, whereas PCaB had significantly increased levels of formate, phenylalanine and pyruvate along with a remarkable decrease in acetoacetate, creatine, glutamine, histidine, isoleucine, leucine and valine when compared to BPH cases. Furthermore, in comparison with PCa, PCaB subjects had higher levels of 3-HB, acetone, citrate, phenylalanine and pyruvate in serum metabolome coupled with lower alanine, creatine, isoleucine, LDL/VLDL, leucine and valine levels. Overall, these results suggested that metabolic changes during the progression and metastasis of PCa are quite different.

**Table 2:** Metabolic changes in serum samples from BPH, PCa, and PCaB

Metabolites (r.u.)	BPH^c^	PCa^d^	PCaB^e^	PCa vs BPH		PCaB vs BPH		PCaB vs PCa	
				p value^a^	FDR^b^	p value	FDR	p value	FDR
3-HB^f^	9.31 ± 5.69	6.18 ± 3.51	8.86 ± 7.11	0.000	0.002	0.701	0.818	0.013	0.022
Acetate	2.11 ± 0.61	1.99 ± 0.63	1.92 ± 0.99	0.250	0.397	0.219	0.348	0.682	0.853
Acetoacetate	2.36 ± 1.19	2.14 ± 0.89	1.94 ± 0.92	0.210	0.397	0.025	0.036	0.245	0.452
Acetone	5.77 ± 4.36	3.21 ± 2.24	5.21 ± 5.21	0.000	0.001	0.528	0.684	0.010	0.042
Alanine	16.80 ± 2.71	18.57 ± 2.52	17.37 ± 3.45	0.000	0.002	0.307	0.413	0.028	0.047
Creatine	2.62 ± 0.62	2.63 ± 0.55	2.36 ± 0.70	0.122	0.330	0.013	0.046	0.026	0.031
Creatinine	1.03 ± 0.32	1.02 ± 0.29	1.00 ± 0.35	0.970	0.970	0.608	0.761	0.632	0.819
Citrate	2.44 ± 0.56	2.34 ± 0.43	2.56 ± 0.61	0.232	0.397	0.137	0.266	0.020	0.031
Formate	0.09 ± 0.04	0.11 ± 0.04	0.12 ± 0.04	0.124	0.330	0.009	0.045	0.204	0.398
Glutamine	18.97 ± 3.20	17.84 ± 2.68	17.70 ± 4.18	0.026	0.047	0.012	0.047	0.829	0.893
Glycine	8.29 ± 1.87	8.01 ± 1.54	8.41 ± 2.48	0.346	0.526	0.771	0.856	0.315	0.550
Gycerol	9.60 ± 1.77	9.50 ± 2.58	10.18 ± 2.75	0.057	0.810	0.178	0.226	0.142	0.193
Histidine	1.20 ± 0.21	1.13 ± 0.18	1.09 ± 0.21	0.022	0.037	0.001	0.035	0.962	0.985
Isoleucine	2.73 ± 0.47	2.72 ± 0.39	2.55 ± 0.43	0.921	0.970	0.012	0.043	0.028	0.035
Lactate	100.63 ± 36.14	92.09 ± 29.81	90.63 ± 33.26	0.040	0.157	0.215	0.348	0.561	0.818
LDL/VLDL	56.61 ± 21.04	61.83 ± 20.69	52.34 ± 23.60	0.147	0.344	0.289	0.404	0.023	0.031
Leucine	13.53 ± 2.02	13.31 ± 1.64	12.63 ± 2.03	0.468	0.655	0.003	0.035	0.005	0.038
Lysine	2.46 ± 0.33	2.54 ± 0.46	2.37 ± 0.45	0.249	0.397	0.235	0.357	0.051	0.137
Phenylalanine	0.68 ± 0.26	0.72 ± 0.31	0.84 ± 0.35	0.078	0.249	0.002	0.028	0.026	0.049
Pyruvate	1.65 ± 0.44	1.65 ± 0.38	1.93 ± 0.56	0.969	0.970	0.002	0.028	0.002	0.034
Threonine	1.76 ± 0.31	1.73 ± 0.40	1.70 ± 0.33	0.623	0.838	0.254	0.370	0.613	0.819
Tyrosine	0.94 ± 0.19	1.02 ± 0.15	1.01 ± 0.33	0.029	0.041	0.180	0.315	0.833	0.893
Valine	9.37 ± 1.47	9.35 ± 1.32	8.65 ± 1.57	0.872	0.970	0.003	0.036	0.034	0.039
α-Glucose	14.19 ± 3.07	14.89 ± 2.89	15.31 ± 3.46	0.177	0.386	0.049	0.167	0.475	0.722
β-Glucose	12.81 ± 3.10	12.98 ± 3.29	12.97 ± 3.45	0.752	0.970	0.782	0.856	0.985	0.985

Data were presented as Mean ± SD

^a^Adjusted by Bonferroni correction across multiple comparisons within each metabolite

^b^Adjusted by false discovery rate (FDR) method across the metabolites within each comparison

^c^BPH, patient with benign prostatic hyperplasia

^d^PCa, prostate cancer

^e^PCaB, prostate cancer with bone metastasis

^f^3-HB, 3-hydroxybutyrate. The colored p value represents significantly decreased (green) or increased (red) from BPH to PCa, BPH to PCaB or PCa to PCaB

### Diagnostic performance of potential metabolic biomarkers

**Fig. 2:**
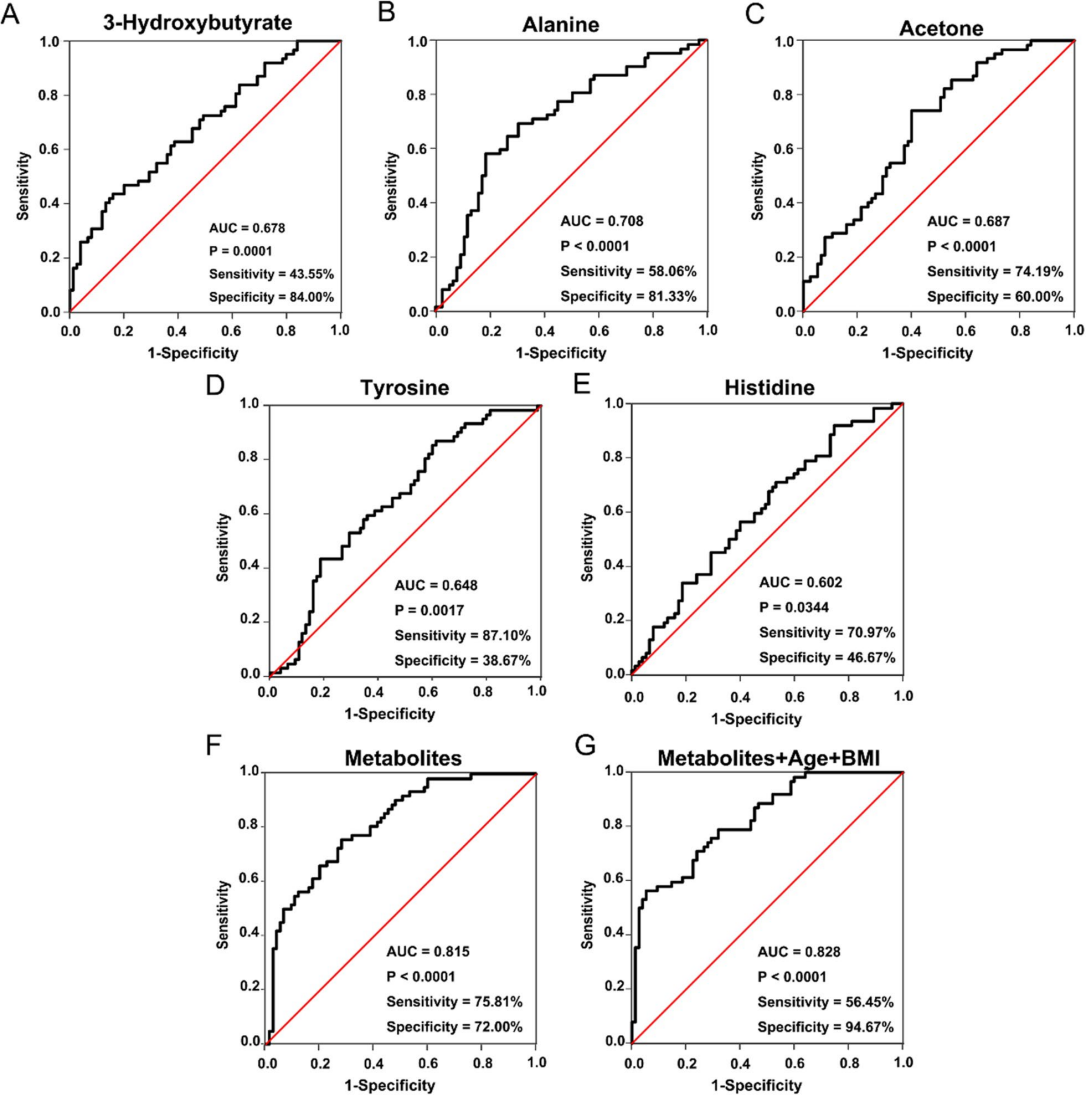
ROC curve analysis of the selected metabolites for discriminating PCa from BPH. The assessment of the diagnostic performance of **A** 3-HB; **B** alanine; **C** acetone; **D** tyrosine; **E** histidine; **F** a combination of five metabolites; **G** a merger of five metabolites with age and BMI

ROC curves analysis, including the AUC value was conducted for evaluating the efficient utilization of the identified significant metabolites for BPH, PCa and PCaB patients screening, based on the metabolites with VIP value > 1 and FDR value < 0.05. Figure 2 illustrates the diagnostic performance of potential metabolic biomarker panels (AUC value > 0.6, p value < 0.05) as they account for discrimination between BPH and PCa groups due to the exhibition of good response by the five metabolite levels that included 3-HB (AUC = 0.678, sensitivity = 43.55%, specificity = 84%), alanine (AUC = 0.708, sensitivity = 58.06%, specificity = 81.33%), acetone (AUC = 0.687, sensitivity = 74.19%, specificity = 60%), tyrosine (AUC = 0.648, sensitivity = 87.10%, specificity = 38.67%) and histidine (AUC = 0.602, sensitivity = 70.97%, specificity = 46.67%). Moreover, the ROC analysis revealed that these five metabolites in combination had a 0.815 AUC value, along with 75.81% and 72% sensitivity and specificity, respectively. While an integration of these five metabolites with age and BMI might achieved 0.828 AUC value coupled with a sensitivity and specificity of 56.45% and 94.67%, respectively.

**Fig. 3:**
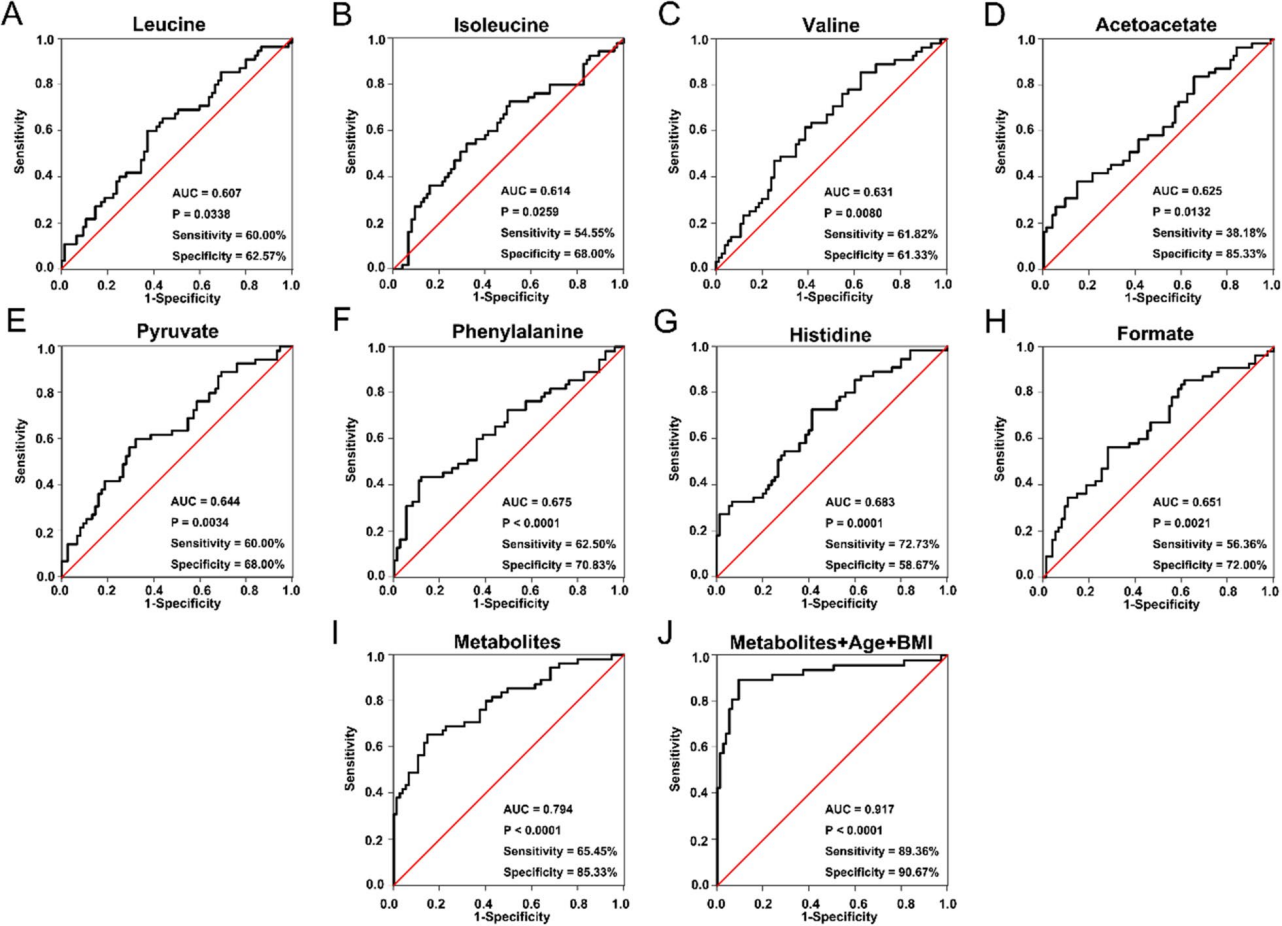
ROC curve analysis of the selected metabolites for differentiating PCaB from BPH. The assessment of the diagnostic performance of **A** leucine; **B** isoleucine; **C** valine; **D** acetoacetate; **E** pyruvate; **F** phenylalanine; **G** histidine; **H** formate; **I** a combination of eight metabolites; **J** a merger of eight metabolites with age and BMI

The ROC analysis results based on the BPH and PCaB differentiating values (AUC value > 0.6 and p value < 0.05) are shown in Fig. 3 which identified eight total of 8 metabolites, that included leucine (AUC = 0.607, sensitivity = 60%, specificity = 62.57%), isoleucine (AUC = 0.614, sensitivity = 54.55%, specificity = 68%), valine (AUC = 0.631, sensitivity = 61.82%, specificity = 61.33%), acetoacetate (AUC = 0.625, sensitivity = 38.18%, specificity = 85.33%), pyruvate (AUC = 0.644, sensitivity = 60%, specificity = 68%), phenylalanine (AUC = 0.675, sensitivity = 62.5%, specificity = 70.83%), histidine (AUC = 0.683, sensitivity = 72.73%, specificity = 58.67%) and formate (AUC = 0.651, sensitivity = 56.36%, specificity = 72%). Moreover, the combination of all these metabolites resulted in a 0.794 AUC value, and the sensitivity and specificity were 65.45% and 85.33%, respectively. Notably, the amalgamation of these eight metabolites with age and BMI was extremely capable of differentiating BPH from PCaB cases, with an AUC value of 0.917, 89.36% sensitivity and 90.67% specificity.

**Fig. 4:**
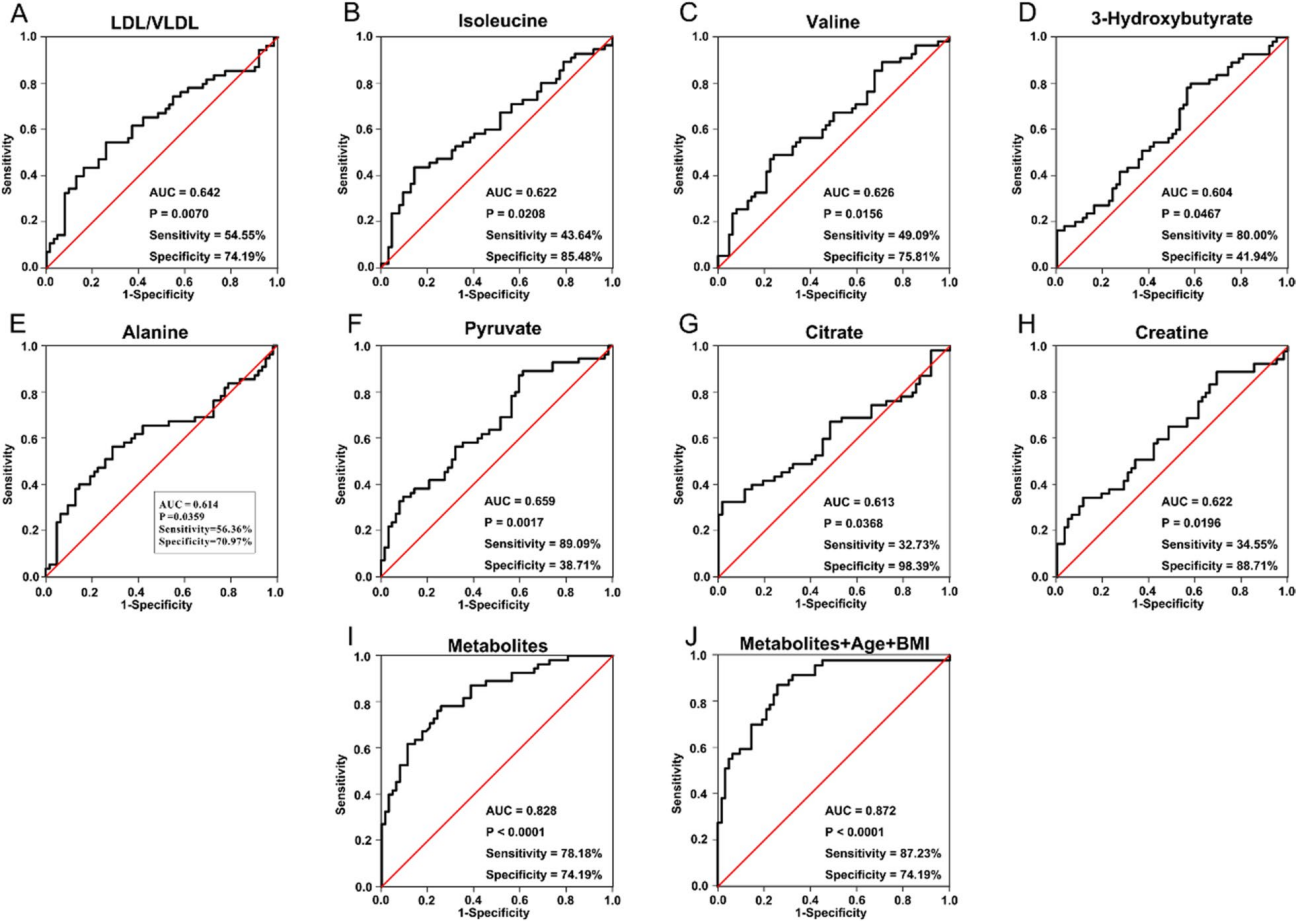
ROC curve analysis of the selected metabolites for discriminating PCaB from PCa. The assessment of the diagnostic performance of **A** LDL/VLDL; **B** isoleucine; **C** valine; **D** 3-HB; **E** alanine; **F** pyruvate; **G** citrate; **H** creatine; **I** a combination of eight metabolites; **J** a merger of eight metabolites with age and BMI

Similarly, the ROC analysis identified top eight metabolites for extricating PCaB diagnosis from PCa as shown in Fig. 4, while their combination exhibited a better classification (AUC = 0.828, sensitivity = 78.18%, specificity = 74.19%) than a singular metabolite: LDL/VLDL (AUC = 0.642, sensitivity = 54.55%, specificity = 74.19%), isoleucine (AUC = 0.622, sensitivity = 43.64%, specificity = 85.48%), valine (AUC = 0.626, sensitivity = 49.09%, specificity = 75.81%), 3-HB (AUC = 0.604, sensitivity = 80%, specificity = 41.94%), alanine (AUC = 0.614, sensitivity = 56.36%, specificity = 70.97%), pyruvate (AUC = 0.659, sensitivity = 89.09%, specificity = 38.71%), citrate (AUC = 0.613, sensitivity = 32.73%, specificity = 98.39%) and creatine (AUC = 0.622, sensitivity = 34.55%, specificity = 88.71%). Additionally, the merging of eight metabolites with age and BMI displayed the best predictability with an AUC value, sensitivity and specificity of 0.872, 87.23% and 74.19%, respectively.

In conclusion, metabolic panels of discriminant metabolites and the amalgamation of metabolites and clinical parameters displayed good diagnostic capability for differentiating among BPH, PCa and PCaB subjects.

### Metabolic pathway analysis of differential metabolites among BPH, PCa, and PCaB

**Fig. 5:**
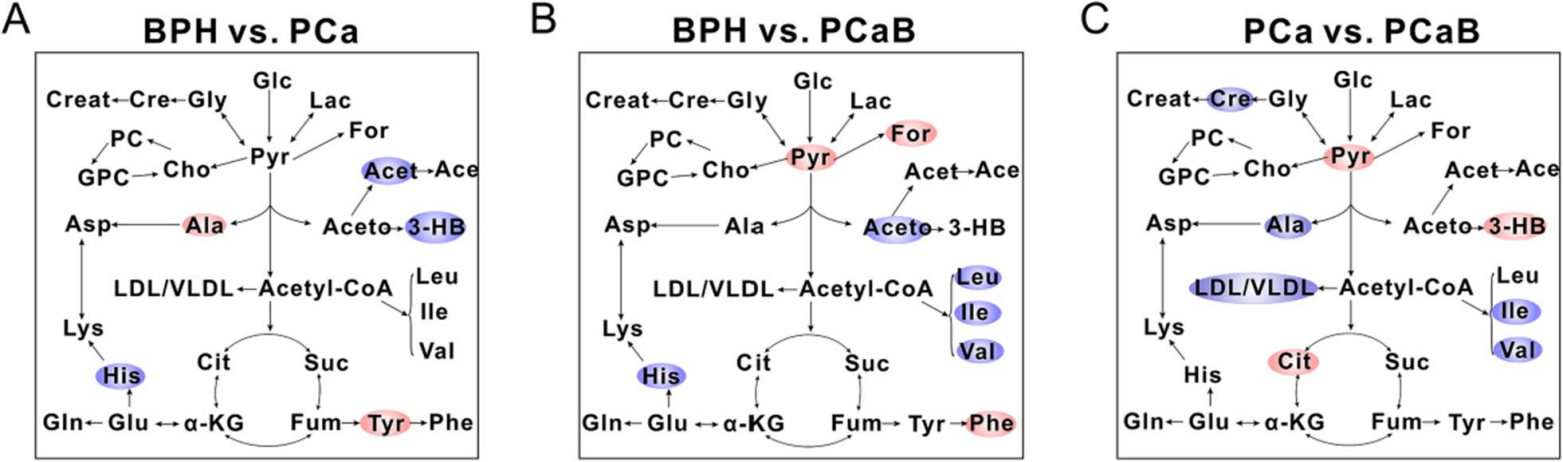
Metabolic pathway analysis. The metabolic pathway was drawn according to the KEGG database and SMPDB database. The red or blue shadings represent the significantly increased or decreased levels of metabolites from BPH to PCa, BPH to PCaB, and PCa to PCaB. Metabolite abbreviation: *3-HB* 3-hydroxybutyrate, *Ace* acetate, *Aceto* Acetoacetate, *Acet* acetone, *Ala* alanine, *Asp* aspartate, *Cho* choline, *Cre* creatine, *Creat* creatinine, *Cit* citrate, *For* formate, *Fum* fumarate, *Glc* glucose, *Glu* glutamate, *Gln* glutamine, *Gly* glycine, *GPC* sn-glycero-3-phosphocholine, *His* histidine, *Ile* isoleucine, *Lac* lactate, *Leu* leucine, *Lys* lysine, *PC* phosphocholine, *Phe* phenylalanine, *Pyr* pyruvate, *Suc* succinate, *Tyr* tyrosine, *Val* valine

Metabolic pathway analysis was carried out based on the differential metabolites for exploring pathway-based metabolic features, as shown in Fig. 5. Our results suggested that a series of metabolic pathways like energy, amino acid, and ketone body metabolism were implicated in metabolic discrimination among BPH, PCa, and PCaB subjects.

## Discussion

Early diagnosis plays a crucial role in the successful treatment for PCa [21]; however, the non-selective use of traditional screening tools for PCa generally leads to overdiagnosis and overtreatment that does not give expected results [22]. Several studies have reported that metabolic disturbances have been associated with increased PCa incidence [23]. Nowadays, metabolomics has emerged as an immensely powerful screening tool in PCa biomarker development due to its inherent advantages, such as noninvasive procedures, high reproducibility, and lower costs [24]. This study examined the metabolic profiles of BPH, PCa, and PCaB serum samples obtained by ^1^H NMR-based metabolomics approach and revealed that serum metabolome analysis exhibited clear discriminations among the three subject groups: BPH, PCa, and PCaB. After conducting univariate statistical and VIP analyses, the most important metabolites were selected, and the diagnostic capacity of these metabolites was evaluated by ROC analysis, while the potential biomarker panels were identified in serum samples for segregating BPH, PCa, and PCaB subjects. Accordingly, the present study identified valuable metabolic panels based on serum metabolome, which exhibited superior performance in discriminating the three groups. A combination of variable metabolites from MRS and MS analysis [12] showed a good classification capacity for discriminating BPH from PCa, with 73.7% of sensitivity, 69% specificity, and 54.1% and 76.3% sensitivity and specificity, respectively. In the present study, significant metabolites in combination for discriminating BPH from PCa patients, had a 0.815 AUC value, along with 75.81% and 72% sensitivity and specificity, respectively. According to Zhang et al. [25], the prognostic value of serum sialic acid levels in predicting PCa and PCaB achieved an AUC value of 0.57, the sensitivity of 60% and the specificity of 58.6%. By combining variable metabolites found in the present study, PCa and PCaB patients were separated with a AUC value of 0.828, a sensitivity and specificity of 78.18% and 74.19%, respectively. Especially, the metabolic biomarker panels in the cluster of differential metabolites and clinical characteristics, showing the AUC values > 0.8, suggesting a relatively good clinical value for diagnosing PCa and its metastases. Based on these, our study imply that serum metabolome based on ^1^H NMR-based metabolomics approach has a great potential as a diagnostic supplemental tool for PCa and its metastases. In our study, it was observed that among all identified potential metabolic biomarkers for different PCa stages, a series of metabolic pathways were mainly involved, including energy, amino acid, and ketone body metabolism. Of note, a series of characteristic metabolic changes were identified, including decreased trends of 3-HB and acetone as well as elevated trends of alanine in PCa patients compared with BPH subjects, while increased levels of 3-HB and acetone as well as decreased levels of alanine in PCaB patients compared with PCa.

Energy metabolism, which expedites the uptake and incorporation of glucose into the biomass needed to produce new cells, is critical for maintaining the abnormal growth and intensive proliferation of cancer cells [26]. Furthermore, the glycolytic breakdown of glucose, the main substrate for energy supply, results in pyruvate production that consequently gets oxidized through the tricarboxylic acid (TCA) cycle for ATP production or converted into lactate by anaerobic glycolysis [27]. According to the Warburg effect [28], most cancer cells primarily convert glucose to lactate through anaerobic glycolysis for meeting the energy requirements for cellular cell proliferation. However, our study revealed that the pyruvate concentration in serum samples was significantly enhanced in PCaB cases compared to PCa and BPH, which might further imply that PCaB may have a reverse Warburg effect in serum metabolome that still needs validation. Citrate is not only an important substrate for de novo lipid synthesis but also serves as a key TCA cycle intermediate for ATP production [29, 30]. In the present study, the citrate level of PCaB serum samples was higher than the PCa samples, which is in line with our previous study [15], that increased TCA cycle intermediates might play a pivotal role in malignant tumor cell growth and proliferation. Additionally, the concentration of creatine, an important regulator of energy metabolism, was remarkably decreased in the PCaB serum levels when compared to PCa and BPH subjects. Given high energy demand of tumor cells [31], a decrease in serum creatine may attributed to providing fuels to meet energy production. Overall, our results implies that a distinct energy metabolism occurred in tumor progression and metastasis.

Out of the many energy sources, amino acids are precisely involved in biosynthesis as well as are important reserves for supporting the survival and proliferation of cancer cells [32]. A recent study investigated the emerging roles of amino acids in epigenetic regulation and initiating immune responses that were related to tumorigenesis and metastatic pathways [33]. Our study noted that a majority of amino acids were increased in the serum samples from BPH to PCa subjects but gradually decreased when the disease progressed to PCaB. It was suggested that leucine, isoleucine, and valine are members of the branched-chain amino acids (BCAAs) that can be catabolized to TCA cycle intermediates for energy production [34], which was also supported by Teahan et al. [35] that BCAAs are potential biomarkers for PCa aggression by using NMR-based metabolomics. Hence, it was evident that increased levels of serum BCAAs are utilized as an energy source for tumor proliferation and development. In contrast, it was also observed that the serum BCAAs levels were significantly decreasing in PCaB when compared with PCa or BPH patients, thereby suggesting that downregulated BCAAs might be closely related to bone metastasis in PCa progression. Recent evidence indicated that histidine catabolism was associated with PCa progression [15] and was consistent with our findings that the histidine level was markedly decreased in the serum levels of both PCa and PCaB patients as compared to BPH patients. As proposed by Lapek et al. in 2015 [36] that histidine phosphorylation might be greatly associated with metastatic PCa. Hence, this concept could be put forth that the downregulation of PCa and PCaB histidine levels, when compared to BPH patients, might be attributed to the upregulated histidine phosphorylation in PCa and the resultant metastasis development. Several previous studies have indicated that alanine level was significantly higher in serum and biopsy tissues when progressing from BPH to PCa [37, 38]. In contrast, a decreased alanine level was associated with advanced cancer stage and poor cancer-specific survival [39]. Accordingly, our study denoted that due to alanine’s differential nature, it was observed that tumor proliferation was consistent with increased protein synthesis; as a result, the alanine serum level was distinctly elevated from BPH to PCa but decreased from PCa to PCaB subjects. Correspondingly, tyrosine and phenylalanine were two additional metabolites that exhibited increased trends in the serum of PCa and PCaB patients. Several studies have demonstrated that dysregulated tyrosine and phenylalanine metabolism is closely related to PCa progression [40, 41]. Another observation in our study was the presence of higher levels of tyrosine and phenylalanine in PCa and PCaB patients’ serum when compared with BPH patients, respectively. It was substantiated by Gomez-Cebrian et al. [42] that phenylalanine hydroxylase (PAH) is the enzyme that metabolizes excess phenylalanine into tyrosine; hence, it is also reported to have a direct association with protein acetylation and energy production [41, 43]. Moreover, our study revealed that a decreased PAH expression might be directly proportional to the enhanced levels of both phenylalanine and tyrosine in PCa and its metastatic progression.

Ketone bodies (3-HB, acetoacetate, and acetone) are alternative mitochondrial energy reserves that can be converted into acetyl-CoA and reutilized as energy substrates[44, 45]. Several studies have stated that ketone body metabolism is critical for tumor biomass expansion [46], as well as the fact that ketogenesis plays an important role in PCa progression [47]. A study by Martinez et al. [48] generated a series of cells and fibroblasts overexpressing the enzyme initiating ketone body production and suggested that the production and reutilization of ketone bodies promote tumor progression and metastasis. A prominent ketone body, 3-HB, has been proved to be specifically associated with metastatic prostate cancer [49]. The current study displayed significant reductions in 3-HB and acetone in PCa cases relative to BPH, but significant elevations were duly observed in PCaB subjects when compared to PCa cases. Collectively, the characteristic changes in 3-HB and acetone levels might be applicable for the potential detection of the PCa progressive stages. However, it was observed that the acetoacetate concentration was significantly decreased in PCaB as compared to BPH patients, which may be due to the uptake of ketone bodies from the tumor tissues in PCa proliferation and development.

Formate is a member of short-chain fatty acids and can be remodeled back for re-synthesizing serine via a one-carbon metabolism pathway [50]. Additionally, formate is an extremely vital component of one-carbon metabolism for tumor proliferation and growth [51, 52]. A study by Meiser et al. [53] revealed that mice with oxidative cancers have higher circulating formate levels than the healthy controls, thereby proposed that elevated formate overflow is a hallmark of oxidative cancers. Our study results also observed an increased formate level in the PCaB patients' serum when compared to BPH, which was also confirmed by our previous study that depicted a higher formate level in the metastatic PCa tissue when compared to other PCa stages [15]. Thus, our results might imply that upregulated formate metabolism mainly occurred in the tumor tissue and serum samples of the tumor in its metastatic stage.

LDL/VLDL is an essential member of lipoproteins, fundamental to the reverse cholesterol transport pathway and lipid homeostasis [54, 55]. Recent studies have demonstrated that lipoprotein might be considered as a risk factor for PCa [56, 57]. However, several literary insights display contradictory findings regarding the association between lipid metabolism and PCa progression and development. A study by Bull et al. [58] suggested there is a weaker association between higher lipoprotein levels and aggressive PCa risk, which was also substantiated by a meta-analysis of 14 large prospective studies that indicated an absence of association between the blood lipoprotein levels and overall PCa or high-grade PCa risk cases [59]. Our results revealed that PCaB cases had lower circulating LDL/VLDL levels than PCa, thus, suggesting that PCaB may have a disruptive lipid metabolism relative to PCa. Moreover, the lipid metabolism inconsistency along with several metabolic aberrations in PCa development could contribute to the initiation of several more studies focused on the extremely significant role of lipid metabolism in PCa progression.

In this study, BPH patients were enrolled as a control group, which is a typical clinical setting. The healthy control donors usually had lower PSA levels and may have a small chance of holding undiagnosed PCa [12]. However, BPH patients with significantly higher levels of PSA are very common among elderly and middle-aged men, which frequently leads to the lack of specificity in PSA measurements. Besides, understanding the metabolic profiles among BPH, PCa and PCaB offers vital information about metabolic biomarkers for PCa progression and its metastases since PSA measurements are not optimal. Moreover, the appropriate use of metabolic biomarkers can provide a more comprehensive evidence for the early screening and diagnosis to predict the corresponding PCa progression and make individualized treatment options. Thus, we believe this is a highly reasonable and relevant clinical setting for diagnosing and classifying of PCa. However, there are several potential limitations in this study: (1) As the majority of BPH patients did not undergo biopsy, there was no relationship analysis between differential metabolic changes and the subjects pathology in this study; but the pathologic analysis of subjects is recommended for elucidating the link of metabolic biomarkers in different PCa pathologies. (2) Although the potential metabolic biomarkers were identified in serum samples, this finding still needs validation by using other biofluids and matching tissues. (3) As our study was a prospective, single-center study with a smaller sample size, large clinical multi-center cohorts would be required for verifying the clinical potential of our findings. (4) Since ^1^H NMR-based metabolomics analysis is generally limited to small metabolites at higher concentrations, multi-omics analysis integrating several useful genes, proteins, and metabolome are recommended for elucidating potential mechanisms underlying the PCa progression and its metastases.

## Conclusion

To conclude, ^1^H NMR-based metabolomics analysis of serum metabolic profiles in BPH, PCa, and PCaB patients can be successfully utilized to establish the significant metabolic signatures related to PCa progression and its metastases. The identified metabolic panels in our study might provide crucial insights into the diagnosis and classification of PCa.

## Supplementary Information

**Additional file 1: Figure S1.** Metabolic changes among different regionsof age and BMI. Principal component analysis (PCA) model was used to examine the metabolic changes among different regionsof (A) age and (B) BMI for detecting the influence of potential covariates on serum metabolome.

## Data Availability

The data used and/or analyzed are available from the corresponding author on a reasonable request.

## Abbreviations

PCa: Prostate cancer
PCaB: PCa patients with bone metastasis
BPH: Benign prostatic hyperplasia
NMR: Nuclear magnetic resonance
BMI: Body mass index
PSA: Prostate-specific antigen
DRE: Digital rectal examination
TRUS: Trans-rectal ultrasound
3-HB: 3-Hydroxybutyrate
