# Supplementary material for: Identification of characteristic metabolic panels for different stages of prostate cancer by 1H NMR-based metabolomics analysis
Source: J Transl Med. 2022 Jun 17;20:275. doi: 10.1186/s12967-022-03478-5 (PMC9205125; doi:10.1186/s12967-022-03478-5)
Supplement: Supplementary file 1 — Additional file 1: Figure S1. Metabolic changes among different regionsof age and BMI. Principal component analysis (PCA) model was used to examine the metabolic changes among different regionsof (A) age and (B) BMI for detecting the influence of potential covariates on serum metabolome. [file 12967_2022_3478_MOESM1_ESM.docx]

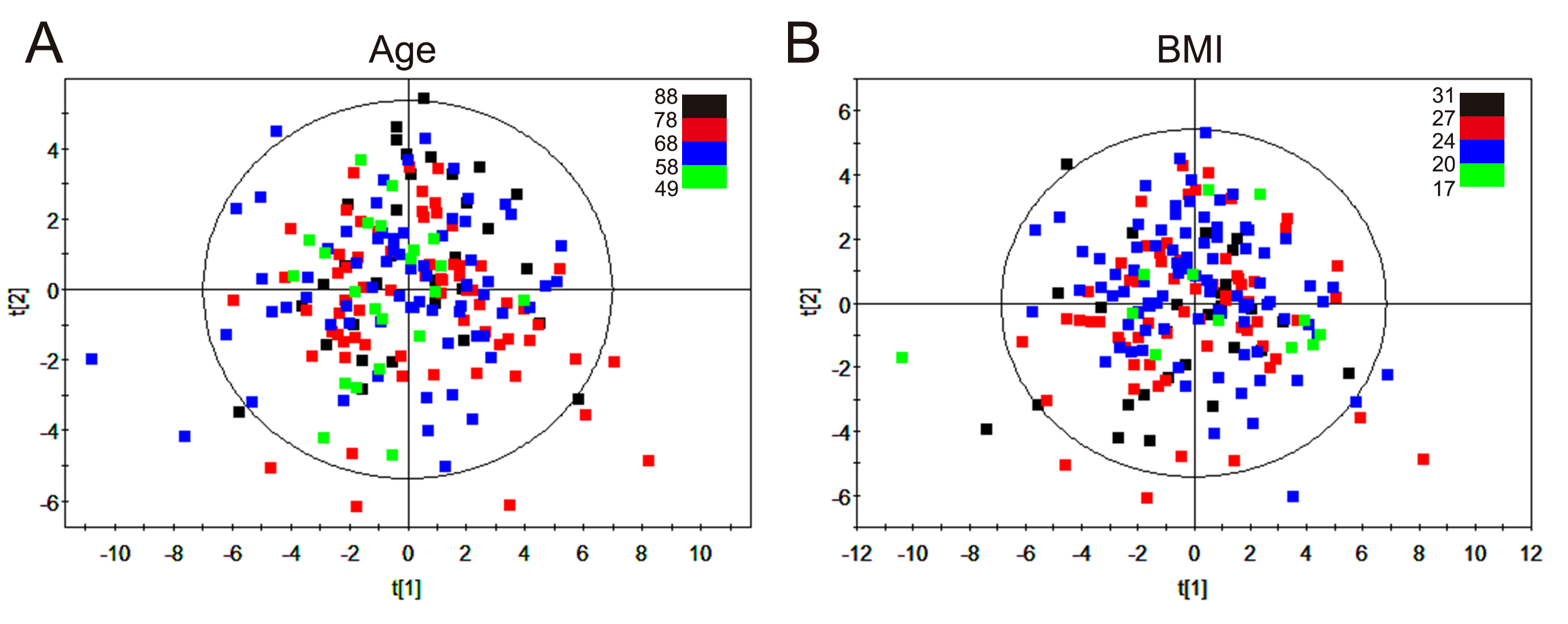


Supplementary Figure 1. Metabolic changes among different regions of age and BMI. Principal component analysis (PCA) model was used to examine the metabolic changes among different regions of (A) age and (B) BMI for detecting the influence of potential covariates on serum metabolome.
